# The Selective NLRP3-inflammasome inhibitor MCC950 Mitigates Post-resuscitation Myocardial Dysfunction and Improves Survival in a Rat Model of Cardiac Arrest and Resuscitation

**DOI:** 10.1007/s10557-021-07282-z

**Published:** 2022-01-01

**Authors:** Guanghui Zheng, Fenglian He, Jing Xu, Juntao Hu, Weiwei Ge, Xianfei Ji, Changsheng Wang, Jennifer L. Bradley, Mary Ann Peberdy, Joseph P. Ornato, Longyuan Jiang, Stefano Toldo, Tong Wang, Wanchun Tang

**Affiliations:** 1 Department of Emergency, Sun Yat-Sen Memorial Hospital, Sun Yat-Sen University, Guangzhou, 510120 China; 2 Weil Institute of Emergency and Critical Care Research, Virginia Commonwealth University, Sanger Hall, 1101 E Marshall St, Box 980266, Richmond, VA 23298- 0279 USA; 3 Institute of Cardiopulmonary Cerebral Resuscitation, Sun Yat-Sen University, Guangzhou, 510120 China; 4 Departments of Internal Medicine and Emergency Medicine, Virginia Commonwealth University Health System, Richmond, 23298 USA; 5 Department of Emergency Medicine, Virginia Commonwealth University Health System, Richmond, 23298 USA; 6 Department of Internal Medicine, Virginia Commonwealth University Health System, Richmond, 23298 USA; 7 Department of Emergency, The Eighth Affiliated Hospital, Sun Yat-Sen University, Shenzhen, 518033 China

**Keywords:** Cardiac arrest, Postresuscitation, NLRP3 inflammasome, MCC950, Cardioprotection

## Abstract

**Purpose:**

To investigate the effects of the selective NLRP3 inflammasome inhibitor MCC950 on post-resuscitation myocardial function and survival in a rat model of cardiopulmonary resuscitation (CPR).

**Methods:**

Thirty-six Sprague Dawley rats were randomized into three groups: (1) MCC950, (2) control, and (3) sham. Each group consisted of a 6 h non-survival subgroup (n = 6) and a 48 h survival subgroup (n = 6). Ventricular fibrillation (VF) was induced and untreated for 6 min. CPR was initiated and continued for 8 min. Resuscitation was attempted with a 4 J defibrillation. MCC950 (10 mg/kg) or vehicle was administered via intraperitoneal injection immediately after the return of spontaneous circulation (ROSC). Myocardial function and sublingual microcirculation were measured after ROSC in the non-survival subgroups. Plasma levels of interleukin Iβ (IL-1β) and cardiac troponin I (cTnI) were measured at baseline and 6 h in the non-survival subgroups. Heart tissue was harvested to measure the NLRP3 inflammasome constituents, including NLRP3, apoptosis-associated speck-like protein (ASC), Caspase-1, and IL-1β. Survival duration and neurologic deficit score (NDS) were recorded and evaluated among survival groups.

**Results:**

Post-resuscitation myocardial function and sublingual microcirculation were improved in MCC950 compared with control (*p* < 0.05). IL-1β and cTnI were decreased in MCC950 compared to control (*p* < 0.01). The MCC950 treated groups showed significantly reduced ASC, caspase-1, and IL-1β compared with the control group (*p* < 0.05). Survival at 48 h after ROSC was greater in MCC950 (*p* < 0.05) with improved NDS (*p* < 0.05).

**Conclusion:**

Administration of MCC950 following ROSC mitigates post-resuscitation myocardial dysfunction and improves survival.

**Supplementary Information:**

The online version contains supplementary material available at 10.1007/s10557-021-07282-z.

## Introduction

Cardiac arrest (CA) remains a significant public health issue. In the United States, the incidence of Emergency Medical Services (EMS)-treated out-of-hospital cardiac arrest (OHCA) in adults is 73 individuals per 100,000. Survival to hospital discharge with good neurologic function after EMS-treated OHCA is only 9.0% [1]. Most survivors from successful cardiopulmonary resuscitation (CPR) die due to severe myocardial and neurologic dysfunction associated with global ischemia/reperfusion (I/R) injury [2]. Currently, only targeted temperature management is strongly recommended for patients with return of spontaneous circulation (ROSC) after cardiac arrest [3]. Exploring novel therapeutics that ameliorate post-resuscitation myocardial function and improve survival outcomes is of great importance.

Evidence shows that inflammatory responses with overproduction of interleukin-1β (IL-1β) play a crucial role in myocardial ischemia/reperfusion (I/R) injury [4–6]. The inflammasome regulates the IL-1β maturation and secretion to the systemic circulation. The nucleotide oligomerization domain (NOD)-like receptor protein-3 (NLRP3) is an intracellular signaling molecule that senses many pathogen-, environmental- and host-derived factors. Upon activation, NLRP3 binds to an apoptosis-associated speck-like protein containing a CARD (ASC). ASC interacts with cysteine protease caspase-1, forming a complex termed the inflammasome. Its activation by danger signals results in the activation of caspase-1, cleaving proinflammatory cytokine IL-1β to its active form and mediates inflammatory cell death pyroptosis [7]. NLRP3 inflammasome activation contributes to regional myocardial I/R injury [8, 9]. Therefore, a potential therapeutic target for treating NLRP3 related diseases is pharmacological NLRP3-inflammasome inhibitors [10–13]. MCC950 is a highly selective NLRP3 inflammasome inhibitor with powerful in vitro and in vivo inhibitory effects [14]. In a mouse model of traumatic brain injury, Ismael et al. found that MCC950 can reduce brain injury [15]. Wang et al. [16] also demonstrated that MCC950 ameliorated lipopolysaccharide-induced lung inflammation in mice. At the same time, van Hout et al. [17] reported that MCC950 reduced infarct size and preserved cardiac function in a pig model of myocardial infarction.

CA and CPR represent systemic I/R injury. In the present study, we investigated the effects of the selective NLRP3 inflammasome inhibitor, MCC950, on post-resuscitation myocardial function and survival in a rat model of CPR.

## Materials and Methods

### Animal Preparation

**Fig. 1:**
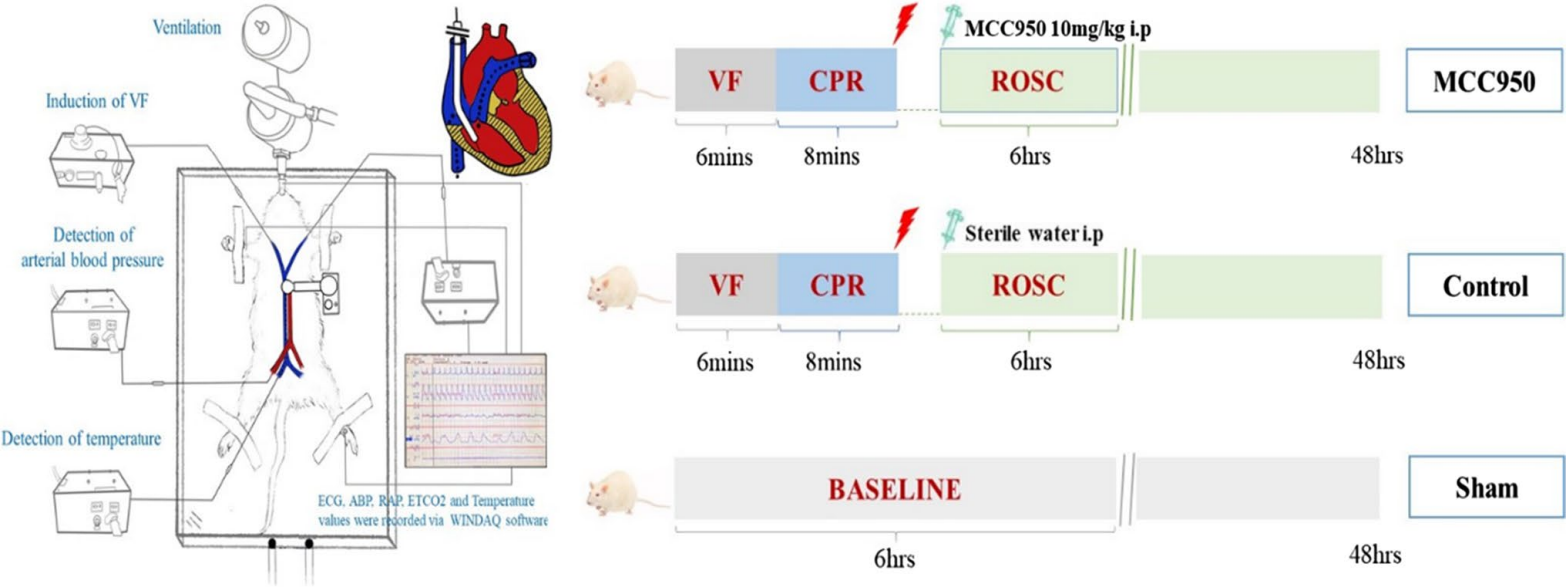
Animal preparation and experimental procedure. After animal preparation, a total of 36 rats were randomized into the sham group, the control group, and the MCC950 group (n = 12). Rats in the control and MCC950 groups underwent 6 min of untreated VF followed by 8 min of CPR and received an IP injection of MCC950 (10 mg/kg dissolved in sterile water according to 10 mg/ml) or sterile water. Rats in the sham group underwent the same surgical procedure without inducing VF and CPR. Each group consisted of a 6 h non-survival subgroup (n = 6) and a 48 h survival subgroup (n = 6). VF, ventricular fibrillation; CPR, cardiopulmonary resuscitation; ROSC, return of spontaneous circulation

Male Sprague Dawley rats, 6–8 months old, weighing 450–550 g, were supplied by a single source breeder (Envigo, Frederick, MD). Animal preparation has been published previously [18–20]. Briefly, animals were fasted overnight and given free access to water. After inhalation of CO_2_, animals were anesthetized by intraperitoneal (IP) injection of pentobarbital (45 mg/kg, Diamondback Drugs, Scottsdale, AZ, USA), and additional doses (10 mg/kg) were administered when needed to maintain anesthesia. The trachea was orally intubated with a 14-gauge cannula mounted on a blunt needle (Abbocath-T; Abbott Hospital Products Division, North Chicago, IL) with a 145° angled tip. End-tidal carbon dioxide (ETCO_2_) was continuously monitored with a side-stream infrared CO_2_ analyzer (Capstar-100 Carbon Dioxide Analyzer; CWE, Ardmore, PA) interposed between the tracheal cannula and ventilator. A polyethylene (PE) tube (PE-50; Becton Dickinson, Sparks, MD) was advanced from the left femoral artery into the descending aorta to measure arterial pressure and blood withdrawal. Another PE tube was advanced through the left external jugular vein into the right atrium to measure right atrial pressure. To detect blood temperature, a thermocouple microprobe (IT-18; Physitemp Instruments, Clifton, NJ) was advanced into the inferior vena cava from the left femoral vein. The temperature was maintained at 37 °C ± 0.5 °C by a heated surgical board. A 3F catheter (Model C-PMS-301 J; Cook Critical Care, Bloomington, IN) was advanced through the right external jugular vein into the right atrium. A pre-curved guidewire was then advanced through the catheter into the right ventricle to induce VF. A conventional lead II electrocardiogram was continuously monitored (Fig. 1). All catheters were flushed intermittently with saline containing 2.5 IU/mL of crystalline bovine heparin. Aminals maintained anesthesia for 6 h observations in the 6 h non-survival subgroup.

### Experimental Procedures

A total of 36 rats were randomized into one of the following three groups: (1) MCC950, rats underwent 6 min of untreated VF followed by 8 min of CPR and received an IP injection of MCC950 (10 mg/kg dissolved in sterile water according to 10 mg/ml, Sigma-Aldrich, St. Louis, MO, USA) after ROSC. (2) Control, rats underwent 6 min of untreated VF followed by 8 min of CPR and received an IP injection of sterile water after ROSC. (3) Sham, rats underwent the same surgical procedure without inducing VF and CPR. Each group consisted of a 6 h non-survival subgroup (n = 6) and a 48 h survival subgroup (n = 6) (Fig. 1). Rats from the 6 h non-survival subgroups were monitored and observed for 6 h post-ROSC and then euthanized for heart tissue harvest. Rats from the 48 h survival subgroups rats were not euthanized at 6 h post-ROSC and were returned to their cage to observe the survival duration and evaluate neurological deficits 48 h after ROSC.

**Fig. 2:**
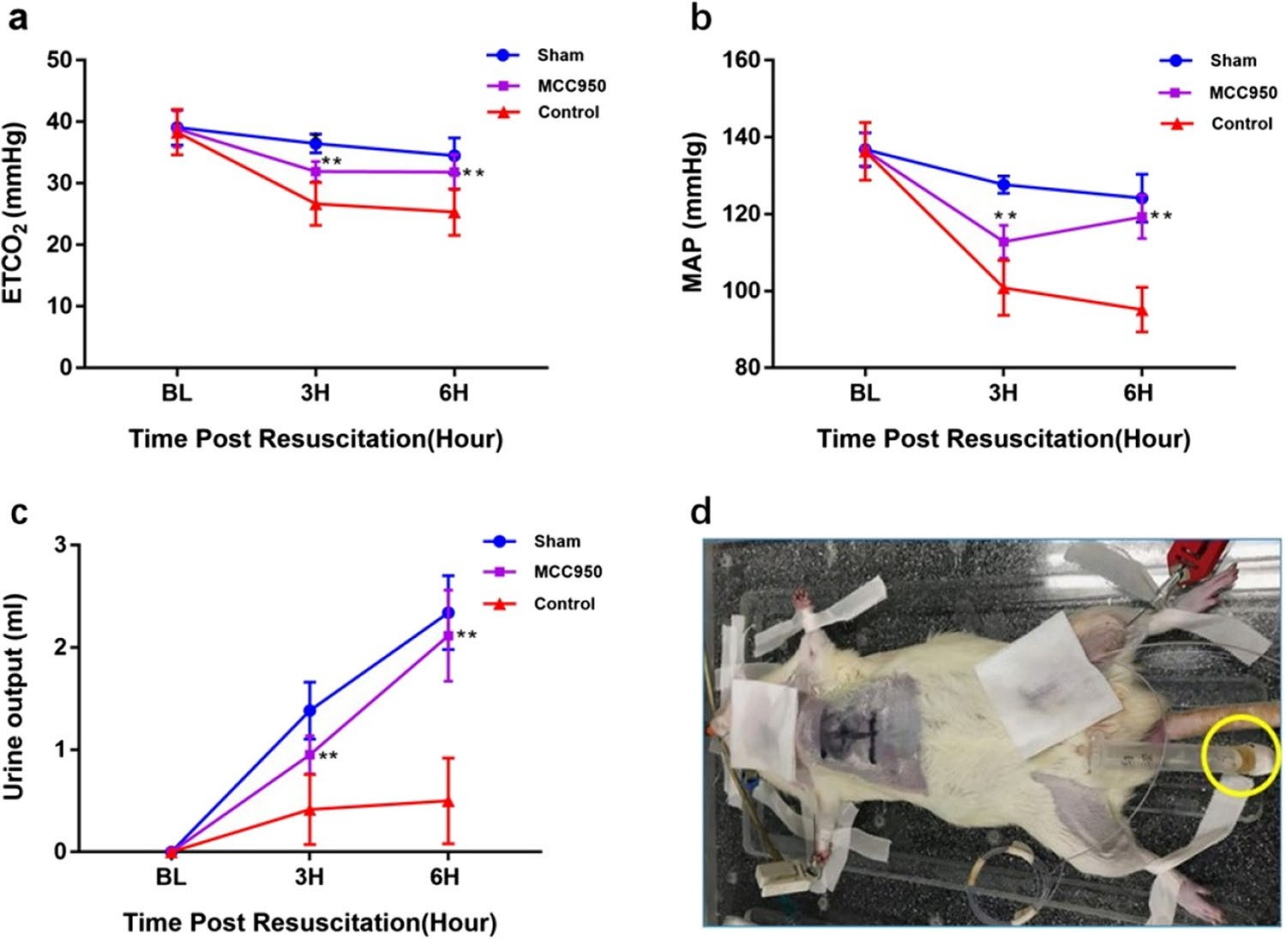
MCC950 increases post-resuscitation ETCO_2_, MAP, and urine output of 6 h non-survival subgroups. **a** ETCO_2_; **b** MAP; **c** Urine output; **d** Diagram of urine collection after ROSC. **p < 0.01 versus the control group with administration of vehicle at ROSC by intraperitoneal injection (MAP and urine output: *p* < 0.0001 for the group effect, time effect, and group*time effect; ETCO_2_: *p* < 0.0001 for the group effect, time effect, and *p* = 0.0083 for group*time effect). ETCO_2_, end-tidal carbon dioxide; MAP, mean arterial pressure

Our established rat model of cardiac arrest and cardiopulmonary resuscitation was utilized [21, 22]. Briefly, 15 min before induction of VF, baseline hemodynamic measurements and echocardiography were obtained. Mechanical ventilation was established at a tidal volume of 0.60 ml/100 g of body weight, 100 breaths/min frequency, and an inspired O_2_ fraction (FiO_2_) of 0.21. Mechanical ventilation was discontinued after the onset of VF. VF was induced through a guidewire, and current flow was continued for 3 min to prevent spontaneous defibrillation. Precordial chest compression together with mechanical ventilation (tidal volume 0.60 ml/100 g body weight, frequency 100 breaths/min, FiO_2_ 1.0) were initiated after 6 min of untreated VF with a pneumatically driven mechanical chest compressor. Precordial chest compression was maintained at a rate of 200/min and synchronized to provide a compression/ventilation ratio of 2:1 with equal compression relaxation for 8 min. Defibrillation was attempted with up to three 4-J counter shocks after 8 min of CPR. ROSC was defined as the return of supraventricular rhythm with a mean aortic pressure above 50 mmHg for 5 min. After ROSC, MCC950 or the same volume of sterile water was administered IP, mechanical ventilation with a FiO_2_ of 1.0 was continued for 1 h, adjusted to 0.5 for the second hour, and 0.21 after that. The animals in 6 h non-survival subgroups were observed and monitored for 6 h after ROSC, which included EtCO_2_, MAP, and urine output. A 3 ml syringe with a curved syringe needle in case of urine leakage was used to collect urine, as shown in Fig. 2d. The urine collection period since the ROSC lasts to 6 h post-ROSC without additional water or fluids administration. Meanwhile, we completed the myocardial function measurements and sublingual microcirculation measurements during the 6 h period. In the 48 h survival subgroup, all catheters in the animals were removed at 4 h ROSC due to the anesthetic plane. Then animals received a subcutaneous injection of Buprenorphine SR Lab (1 mg/kg, ZooPharm, Laramie, WY, USA) and were returned to their cage without monitoring EtCO_2_, MAP, and urine output. Euthanasia occurred with an intravenous (IV) injection of Euthsol (150 mg/kg). The left ventricle was then rapidly harvested and frozen in liquid nitrogen for immunoblotting.

### Hemodynamics Measurements

Electrocardiogram, aortic and right atrial pressures, ETCO_2_, and blood temperature were continuously recorded on a personal computer-based data-acquisition system supported by WINDAQ software (DATAQ, Akron, OH, USA). Coronary perfusion pressure (CPP) was calculated as the difference in time-coincident diastolic aortic and right atrial pressures and displayed in real-time. Urine output was recorded in the non-survival subgroups.

### Myocardial Function Measurements

Ejection fraction (EF), cardiac output (CO), and myocardial performance index (MPI) at baseline, 1 h, 3 h, and 6 h after ROSC were measured by echocardiography (HD11XE; Philips Medical Systems, Eindhoven, Netherlands, USA) with a 12.5 HZ transducer in the 6 h non-survival subgroups. CO and EF were adopted to estimate myocardial contractility. MPI combines time intervals related to systolic and diastolic functions and reflects the global cardiac function calculated using the formula (a–b)/b, where a = mitral closure-to-opening interval (time interval from cessation to onset of mitral inflow) and b = ET (aortic flow ejection time, obtained at the LV outflow tract) [23]. All measurements were reviewed and confirmed separately by two blinded investigators.

### Sublingual Microcirculation Measurement

Sublingual microcirculation was visualized at 6 h after ROSC within the non-survival groups by a sidestream dark-field (SDF) imaging device (MicroScan; MicroVision Medical Inc., Amsterdam, Netherlands) with a 5 × imaging objective, resulting in an on-screen magnification of 276x. Three discrete fields for each were captured to minimize motion artifacts. Microvascular images were recorded on a DVD with a DVD recorder (DMR-EZ47V; Panasonic AVC Networks, Dalian, China). Microvascular flow index (MFI) was quantitated by Pozo et al. [24]. The image was divided into four quadrants, and the predominant type of blood flow (absent = 0, intermittent = 1, sluggish = 2, and normal = 3) was assessed in the small vessels (smaller than 20 µm in diameter, representing primarily the capillaries) of each quadrant. The MFI score represented the average values of the four quadrants. The proportion of perfused vessels (PPV) and perfused vessel density (PVD) was measured based on the method of De Backer et al. [25]. Vessel density was calculated as the number of vessels crossing the catheters divided by the total length of the catheters. All recordings were analyzed by two blinded independent observers.

### ELISA Analysis

One milliliter blood samples were collected at baseline and 6 h post-ROSC in the 6 h non-survival subgroups. After centrifugation (3000 × g, 15 min, 4 °C), plasma was removed and stored at −80 °C for further enzyme-linked immunosorbent assay (ELISA) analysis. Protein levels of cardiac troponin I (cTnI) and IL-1β were quantified using a commercial cTnI ELISA kit (LS-F23616, LifeSpan BioSciences, Seattle, WA, USA) and an IL-1β ELISA kit (RLB00, R&D Systems, Minneapolis, MN, USA). ELISA measurement was performed following the manufacturer’s instructions.

### Western Blot Analysis

Heart tissue total protein was extracted using  a NE-PER Nuclear and Cytoplasmic Extraction Reagent kit (78,833, Thermo Scientific, Waltham, MA, USA) and performed according to the manufacturer’s instructions. Homogenized heart tissue was centrifuged at 12,000 rpm for 10 min at 4 °C, and supernatants were obtained. Protein concentration was determined by a Bio-Rad Protein Assay kit II (500–0002, BIO-RAD, Irvine, CA, USA). Western blot analysis was performed following the General V3 Western Workflow Blotting Protocol (Bulletin 6390, BIO-RAD, Irvine, CA, USA). Primary antibodies against NLRP3, caspase-1(p20), IL-1β (1:1000; ab214185, 1:1000; ab1872, 1:2500; ab9722, Abcam, MA, USA) and ASC (1:500; sc-514414, Santa Cruz, CA, USA) were used. Protein bands were imaged with the ChemiDoc MP Imaging System. Band densities were quantified and normalized using Image Lab™ Software (1,709,690, BIO-RAD, Irvine, CA, USA).

### Measurement of Survival and Neurological Deficit Score (NDS)

Level of consciousness, brain stem function, and overall performance were evaluated and scored according to the method of Hendrickx HH, which ranged from 0 (no observed neurologic dysfunction) to 500 (death or brain death) [26]. Survival duration was recorded, and neurologic deficit score was evaluated at 48 h intervals after ROSC. The NDSs was assessed and confirmed by two blinded investigators.

### Statistical Analysis

All data are reported as mean ± SD. Statistical analysis was performed with SPSS 22.0 software and GraphPad Prism 7. For measurements among groups, one-way analysis of variance and Tukey multiple-comparison tests were performed for normally distributed data, while a Kruskal–Wallis test was applied for the non-normally distributed data, and then a Bonferroni test was performed for the multiple comparisons. Analysis of repeated-measurement two-way analysis of variance (ANOVA) and Tukey multiple-comparison techniques were performed to determine differences between time-based measurements within each group. Log-rank (Mantel-Cox) test was used for survival analysis. A value of *p* < 0.05 was considered statistically significant.

## Results

### Baseline Physiologic Parameters and CPR Characteristics

All 36 animals utilized were successfully resuscitated. There were no differences in body weight, blood temperature, EtCO_2_, hemodynamics, and myocardial function (EF, CO, and MPI) at baseline among groups. There were no differences in CPP during CPR or in the number of defibrillations required to ROSC among groups (Table 1).

**Table 1:** Baseline physiological parameters and CPR characteristics in groups

Variables	Sham group	Control group	MCC950 group	*P* value
Body weight (g)	493.42 ± 7.35	493.54 ± 11.22	497.18 ± 8.99	NS
Blood temperature (°C)	36.78 ± 0.48	36.91 ± 0.32	37.07 ± 0.32	NS
Heart rate (beats/min)	372.83 ± 10.23	373.92 ± 11.05	376.33 ± 11.48	NS
ETCO_2_ (mmHg)	40.07 ± 3.30	39.16 ± 3.60	39.13 ± 2.57	NS
RAP (mmHg)	0.76 ± 1.41	0.36 ± 1.67	0.83 ± 1.63	NS
MAP (mmHg)	136.75 ± 6.20	137.92 ± 5.57	138.08 ± 6.37	NS
EF	0.729 ± 0.15	0.730 ± 0.12	0.731 ± 0.14	NS
CO (L/min)	0.17 ± 0.05	0.18 ± 0.01	0.18 ± 0.01	NS
MPI	0.70 ± 0.05	0.67 ± 0.06	0.70 ± 0.71	NS
Plasma IL-1β (pg/ml)	13.00 ± 5.76	13.50 ± 1.87	14.50 ± 4.89	NS
Plasma cTnI (pg/ml)	55.00 ± 5.66	50.00 ± 12.73	57.50 ± 7.78	NS
CPP in PC2(mmHg)	–	31.42 ± 1.73	31.67 ± 2.10	NS
CPP in PC4 (mmHg)	–	28.33 ± 1.23	28.08 ± 1.38	NS
CPP in PC6 (mmHg)	–	27.50 ± 1.09	27.17 ± 1.27	NS
CPP in PC8 (mmHg)	-	25.83 ± 1.59	25.67 ± 1.72	NS
Numbers of DF(n)	–	1.33 ± 1.03	1.58 ± 0.79	NS

ETCO_2_, end-tidal CO_2_; RAP, right atrial pressure; MAP, mean arterial pressure; EF, NS ejection fraction; CO, cardiac output; MPI, myocardial performance index; CPP, coronary perfusion pressure; IL-1β, interleukin 1β; cTnI, cardiac troponin I; PCn, N minute after precordial compression; DF, defibrillation; NS, no significance

### MCC950 increases post-resuscitation ETCO_2_, MAP, and urine output

After resuscitation, the MAP, ETCO_2_, and urine output were significantly decreased in the control group compared to the sham group at 6 h post-ROSC (MAP 95.8 ± 4.5 vs. 124.2 ± 6.2 mmHg, *p* < 0.01; ETCO_2_ 25.3 ± 3.8 vs. 34.5 ± 2.9 mmHg, *p* < 0.01; urine output 0.5 ± 0.4 vs. 2.2 ± 0.4 ml, *p* < 0.01). While the MAP, ETCO_2_, and urine output were significantly higher in the MCC950 group compared with the control group at 6 h post-ROSC (MAP 119.3 ± 5.7 vs. 95.8 ± 4.5 mmHg, *p* < 0.01; ETCO_2_ 30.6 ± 5.0 vs. 25.3 ± 3.8 mmHg, *p* < 0.01; urine output 2.1 ± 0.4 vs. 0.5 ± 0.4 ml, *p* < 0.01) (Fig. 2a–d).

### MCC950 reduces the severity of post-resuscitation myocardial dysfunction

**Fig. 3:**
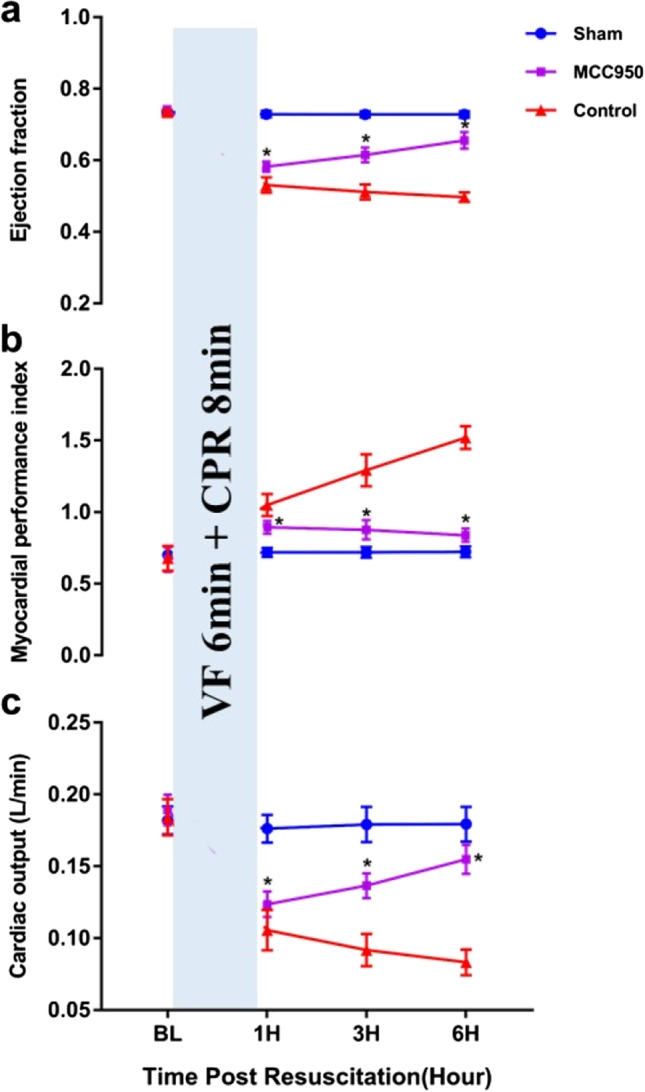
MCC950 improves post-resuscitation myocardial performance index, ejection fraction, and cardiac output. *p < 0.05 versus control with administration of sterile water at ROSC by intraperitoneal injection (*p* < 0.0001 for the group effect, time effect, and group*time effect). BL, baseline; VF, ventricular fibrillation; CPR, cardiopulmonary resuscitation; H, hour; ROSC, return of spontaneous circulation

There was no significant difference in myocardial function (EF, CO, and MPI) among the three groups at baseline (Table 1). However, EF (MCC950 vs. sham 0.6 ± 0.01 vs. 0.7 ± 0.01, *p* < 0.05; control vs. sham 0.5 ± 0.02 vs. 0.7 ± 0.01, *p* < 0.05), CO (MCC950 vs. sham 0.1 ± 0.01 vs. 0.2 ± 0.01 L/min, *p* < 0.05; control vs. sham 0.1 ± 0.01 vs. 0.2 ± 0.01 L/min, *p* < 0.05), and MPI (MCC950 vs. sham 0.9 ± 0.04 vs. 0.7 ± 0.03, *p* < 0.05; control vs. sham 1.1 ± 0.07 vs. 0.72 ± 0.03, *p* < 0.05) were significantly impaired in all successfully resuscitated animals at the 1 h post ROSC when compared with the sham animals. Impairment of EF (MCC950 vs. control 0.6 ± 0.02 vs. 0.5 ± 0.02, *p* < 0.05), CO (MCC950 vs. control 0.14 ± 0.01 vs. 0.09 ± 0.01 L/min, *p* < 0.05), and MFI (MCC950 vs. control 0.9 ± 0.07 vs. 1.3 ± 0.10, *p* < 0.05) were improved at 3 h post ROSC in the MCC950 group compared to the control. Significant improvement of myocardial function with a much higher EF (MCC950 vs. control 0.7 ± 0.02 vs. 0.5 ± 0.02, *p* < 0.05), CO (MCC950 vs. control 0.2 ± 0.01 vs. 0.1 ± 0.01 L/min, *p* < 0.05) and lower MPI (MCC950 vs. control 0.8 ± 0.05 vs. 1.5 ± 0.07, *p* < 0.05) was observed after 6 h of ROSC in the animals treated with MCC950 compared with the control animals (Fig. 3).

### MCC950 improves post-resuscitation sublingual microcirculation

**Fig. 4:**
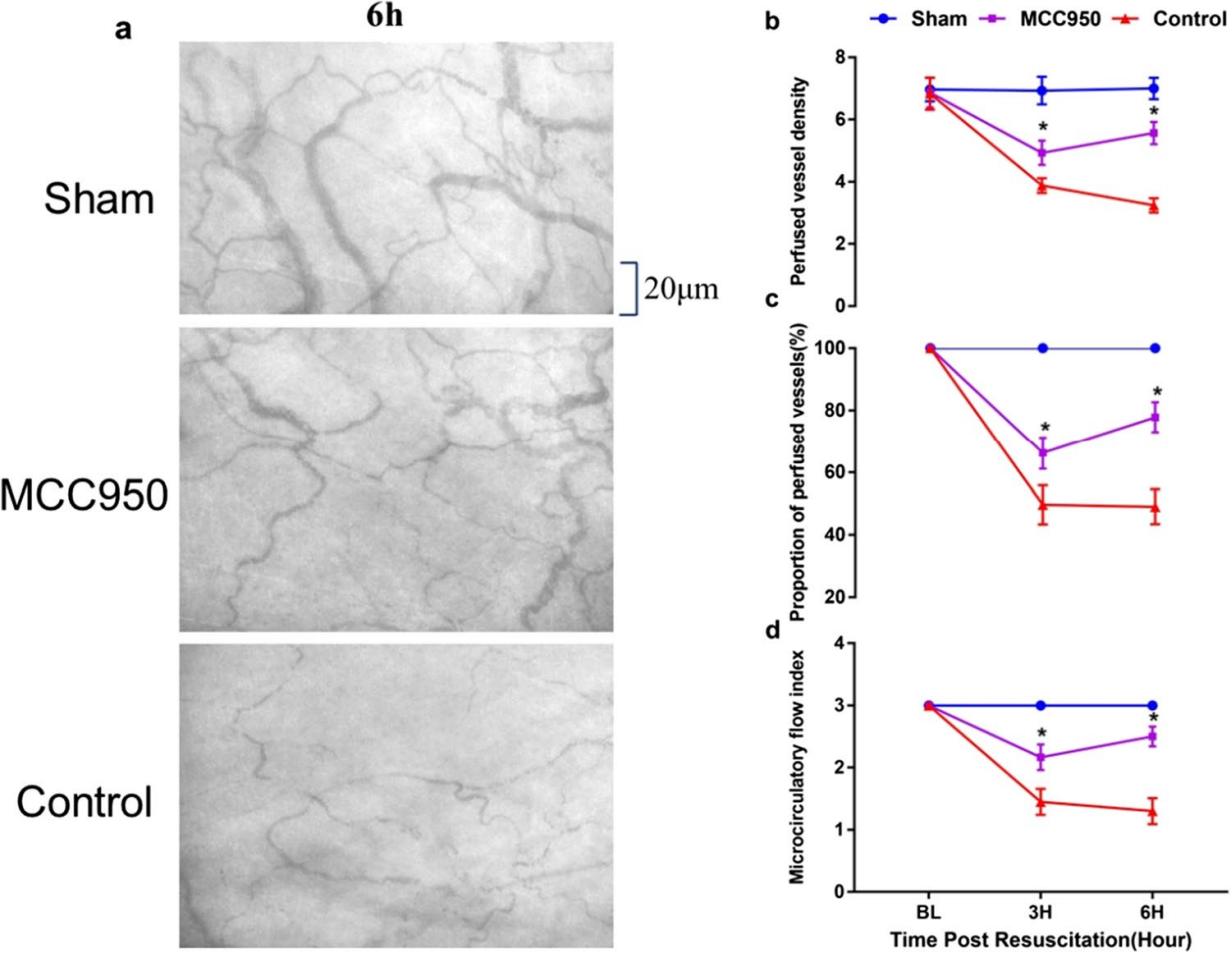
Effect of MCC950 on sublingual microcirculation indicated by PVD, PPV, and MFI at 6 h post-ROSC. MCC950 significantly improved sublingual microcirculation. *p < 0.05 versus the control group with administration of vehicle at ROSC by intraperitoneal injection (*p* < 0.0001 for the group effect, time effect, and group*time effect). PVD, perfused vessel density; MFI, microcirculatory flow index; PPV, proportion of perfused vessels; ROSC, return of spontaneous circulation

Sublingual microcirculation indicated by MFI, PPV, and PVD is summarized in Fig. 4b–d. After successful resuscitation, MFI, PPV, and PVD were impaired significantly in all CPR animals compared to sham at 3 h, 6 h post-ROSC (*p* < 0.05). However, significantly less impairment of MFI (2.2 ± 0.2 vs. 1.5 ± 0.2, *p* < 0.05), PPV (66.2 ± 5.0 vs. 50.8 ± 6.3, *p* < 0.05), and PVD (4.9 ± 0.4 vs. 3.9 ± 0.2, *p* < 0.05) was observed in animals treated with MCC950 when compared to the control animals at 3 h post ROSC. All animals treated with MCC950 exhibited a much better sublingual microcirculation with significantly higher MFI (2.5 ± 0.2 vs. 1.3 ± 0.2, *p* < 0.05), PPV (77.9 ± 4.9 vs. 49.3 ± 5.0, *p* < 0.05), and PVD (5.6 ± 0.4 vs. 3.3 ± 0.3, *p* < 0.05) than the control animals after 6 h of ROSC. Examples of the images of sublingual microvasculature images obtained by the side-stream dark-field imaging device at 6 h post-ROSC are shown in Fig. 4a.

### MCC950 improves post-resuscitation 48 h survival rate, neurologic deficit

**Fig. 5:**
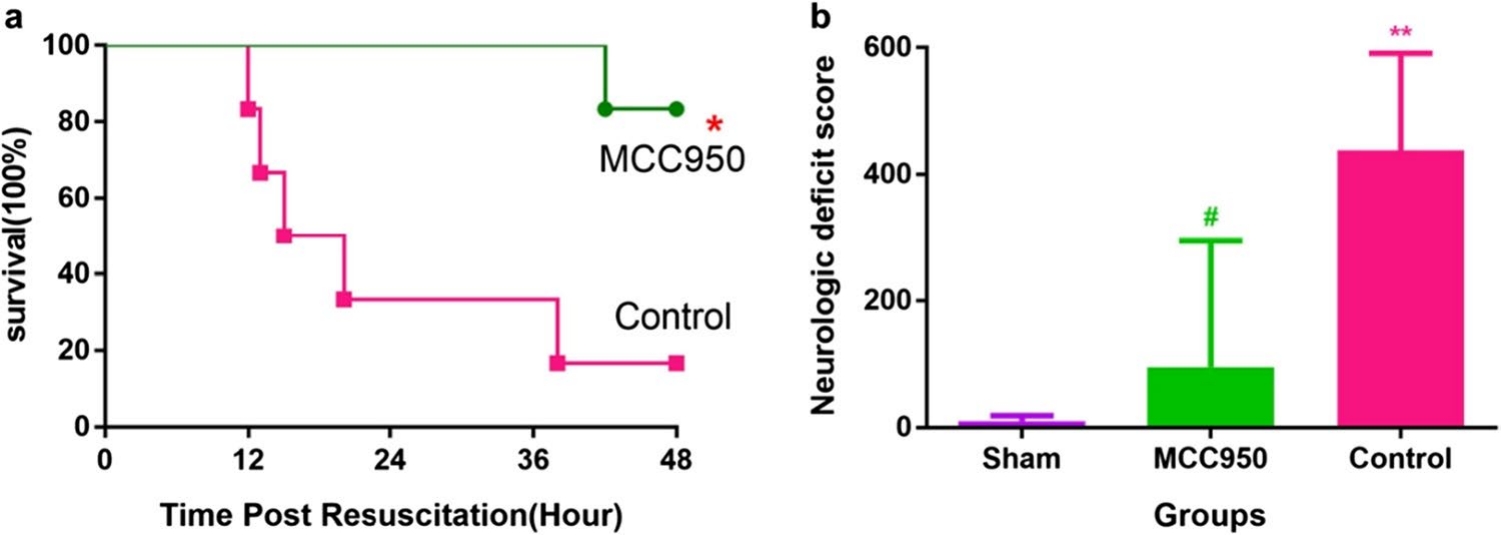
MCC950 improves post-resuscitation 48 h survival duration and neurologic deficit. **a** 48 h survival curves; **b** NDS at ROSC 48 h. *p < 0.05 versus control (administration of vehicle at ROSC by intraperitoneal injection); **p < 0.01 versus sham (underwent the same surgical procedures without VF and CPR); #*p* < 0.05 versus control. NDS, neurological deficit score; ROSC, return of spontaneous circulation

Overall survival rate at 48 h after ROSC was greater in MCC950 compared to the control (5/6 vs. 1/6, *p* < 0.05) (Fig. 5a). Five of 6 rats treated with MCC950 survived over the 48 h with an average duration of survival of 47.0 ± 2.5 h compared with 1 of 6 rats that successfully survived for 48 h with an average duration of survival of 24.3 ± 15.1 h in the control group. NDS at 48 h after ROSC was also improved in MCC950 compared to the control (95 ± 198 vs. 438 ± 153, *p* < 0.05) (Fig. 5b, Suppl.video1-3).

### MCC950 decreases post-resuscitation IL-1β and cTnI plasma levels

**Fig. 6:**
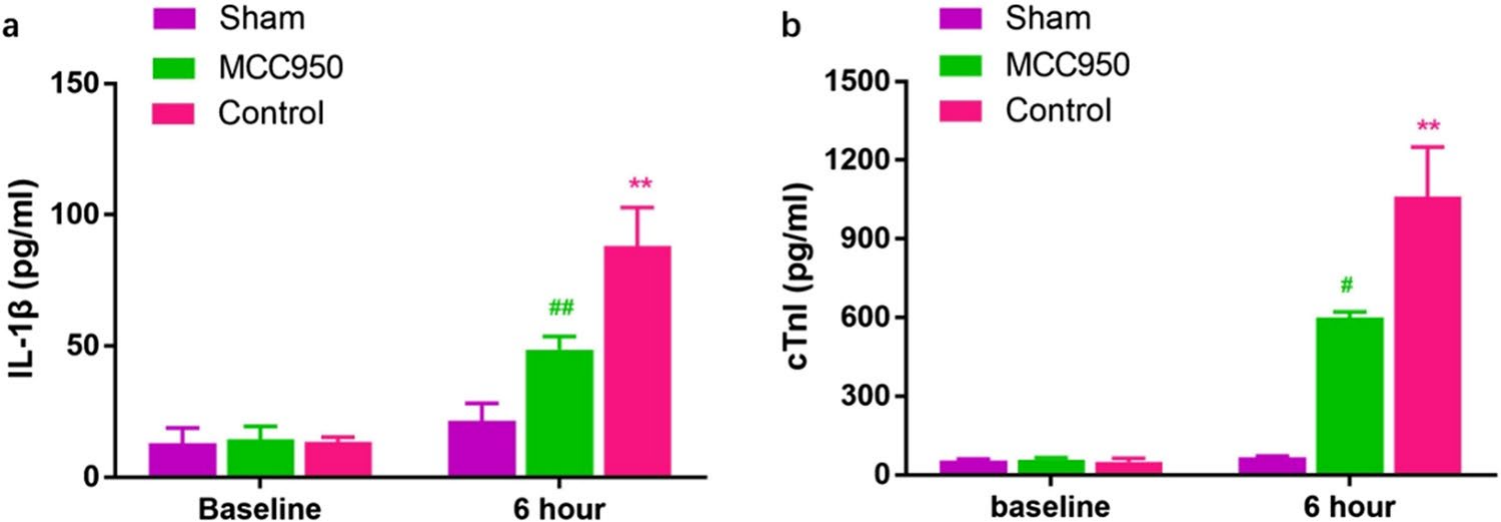
Effect of MCC950 on the expression of IL-1β and cTnI levels in the plasma of 6 h non-survival subgroups at different times. **a** Levels of IL-1β; **b** Levels of cTnI. Post-resuscitation levels of IL-1β and cTnI are significantly increased. MCC950 treatment post-ROSC reduces levels of IL-1β and cTnI. **p < 0.01 versus sham (underwent the same surgical procedures without VF and CPR); ##*p* < 0.01 versus control; # *p* < 0.05 versus control (administration of vehicle at ROSC by intraperitoneal injection). IL-1β, interleukin-1 beta; cTnI, cardiac troponin I; VF, ventricular fibrillation; CPR, cardiopulmonary resuscitation; ROSC, return of spontaneous circulation

There were no differences in IL-1β and cTnI plasma levels at baseline (Table 1). IL-1β plasma levels (MCC950 vs. sham 48.7 ± 5.0 vs. 21.5 ± 6.6 pg/ml, *p* < 0.01; control vs. sham 88.2 ± 14.7 vs. 21.5 ± 6.6 pg/ml, *p* < 0.01) were significantly increased at ROSC 6 h in the MCC950 group and control group compared to sham. However, MCC950 significantly reduced plasma IL-1β (48.7 ± 5.0 vs. 88.2 ± 14.7 pg/ml, *p* < 0.01) compared with the control group. Meanwhile, the concentration of plasma cTnI (MCC950 vs. sham 600.5 ± 21.9 vs. 66.5 ± 5.0 pg/ml, *p* < 0.05; control vs. sham 1060.0 ± 190.9 vs. 66.5 ± 5.0 pg/ml, *p* < 0.01) was also markedly increased in control and MCC950 at 6 h post ROSC compared with sham. However, MCC950 significantly reduced plasma cTnI (600.5 ± 21.9 vs. 1060.0 ± 190.9 pg/ml, *p* < 0.05) compared to control (Fig. 6a, b).

### MCC950 decreases expression levels of the principal constituents of NLRP3 inflammasome within the left ventricle

**Fig. 7:**
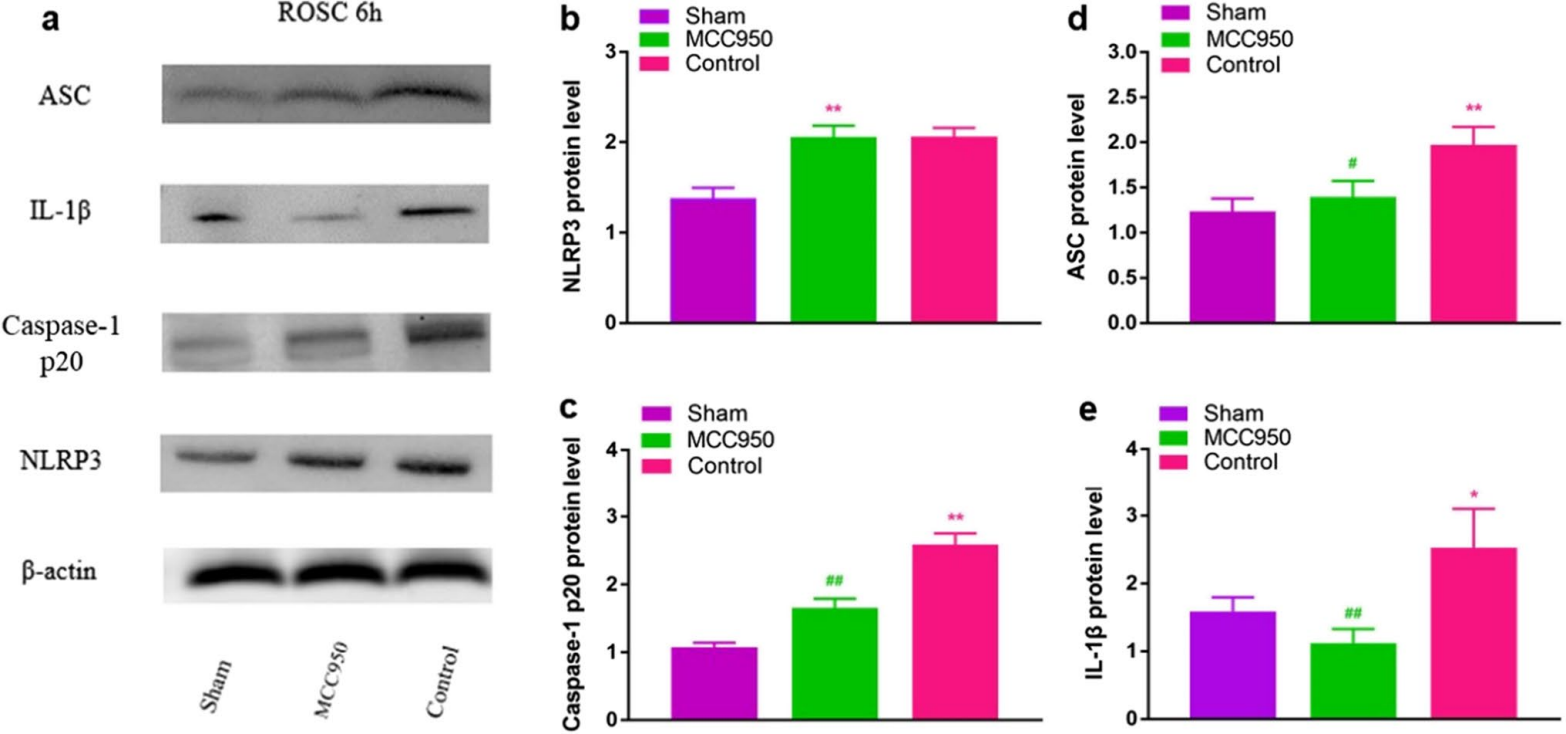
Effect MCC950 on the expression of principal constituents of NLRP3 inflammasome in left ventricles of 6 h non-survival subgroups. **a** Representative and quantitative analysis of **b** NLRP3, **c** Caspase-1, **d** ASC, and **e** IL-1b expression demonstrates corresponding differences in the left ventricle at 6 h post-ROSC. MCC950 treatment post-ROSC reduces levels of ASC and subsequent caspase-1 and IL-1β maturation. *p < 0.05 versus sham (underwent the same surgical procedures without VF and CPR); **p < 0.01 versus sham; #*p* < 0.05 versus control (administration of vehicle at ROSC by intraperitoneal injection); ##*p* < 0.01 versus control. ROSC, return of spontaneous circulation; NLRP3, nucleotide oligomerization domain (NOD)-like receptor protein-3; ASC, apoptosis associated speck-like; IL-1β, interleukin-1 beta

Expression levels of NLRP3, ASC, caspase-1 p20, and IL-1β were significantly increased in MCC950 and control compared to sham (*p* < 0.05). However, the MCC950 treated group showed a significant decrease in ASC, caspase-1 p20, and IL-1β compared to control (*p* < 0.05). MCC950 had no influence on increased NLRP3 protein after post-resuscitation (Fig. 7a–e).

## Discussion

In the present study, our findings indicate that NLRP3 inflammasome activation mediated overproduction of IL-1β is involved in post-resuscitation myocardial dysfunction in a rat model of CA and CPR. We further demonstrated that administration of the selective NLRP3-inflammasome inhibitor MCC950 following ROSC reduces circulatory IL-1β plasma level, mitigates post-resuscitation myocardial dysfunction, and improves 48 h survival rate together with better neurological function in a rat model of CA and CPR.

Global ischemia and reperfusion injury after cardiac arrest and successful resuscitation is associated with myocardial and cerebral dysfunctions and a high post-resuscitation mortality rate [27, 28]. In line with preclinical and clinical studies, the control rats had a lower survival rate, and only one rat survived 48 h post-ROSC accompanied by a more significant neurologic deficit. Cardiac function was significantly impaired in control animals after ROSC. Plasma cTnI levels indicating myocardial injury were significantly increased in control animals. The ejection fraction and cardiac output were significantly decreased in the control compared to sham. Previous studies have demonstrated that MAP, ETCO_2_, sublingual microcirculation, and urine output are closely related to cardiac output [29]. Our research also found that the control rats that underwent CA and CPR had a significant decrease in MAP, ETCO2, urine output, and poor sublingual microcirculation due to the reduction in cardiac output, which could predict an outcome.

Inflammatory responses with over-production of IL-1β are well documented to play a significant role in the pathophysiology of myocardial I/R injury [4, 30]. The NACHT, LRR, and PYD domains-containing protein 3 (NLRP3) inflammasome is an intracellular polyprotein complex that is well documented for its capacity to control the proteolytic activation of caspase-1; subsequently, activated caspase-1 induces the production of mature interleukin-1β (IL-1β) and the inflammatory cell death process is known as pyroptosis [7]. Ischemia/reperfusion injury triggers a sterile inflammatory reaction. Recently, growing evidence suggests that NLRP3 inflammasome activation-mediated pyroptosis contributes to regional myocardial ischemia–reperfusion injury, which leads to myocardial dysfunction. In several acute myocardial infarction models, both IL-1β signaling and pyroptosis induce amplification of the initial ischemic damage, lead to the expansion of the infarct size, and decrease cardiac contractility and cardiac output [9, 31]. In the current study, we further demonstrated that control rats had high IL-1β plasma levels. The myocardial dysfunction was accompanied by the elevation of NLRP3, ASC, Caspase-1, and IL-1β in the myocardium. Our findings indicate that NLRP3 inflammasome activation following global myocardial I/R injury was involved in post-resuscitation myocardial dysfunction in a rat model of CA and CPR. NLRP3 inflammasome activation follows both global myocardial I/R injury and local myocardial I/R injury.

Several studies indicate that inhibition of the NLRP3 inflammasome possesses cardioprotective effects in different cellular and animal models. MCC950 is a small molecular compound that selectively inhibits NLRP3 inflammasome activation and subsequent production of IL-1β both in vitro and in vivo [14]. Studies have shown that MCC950 exerts protective effects in regional ischemia and reperfusion models. van Hout et al. [15] reports that MCC950 reduces infarct size and preserves cardiac function in a randomized, blinded translational large animal MI model. We measured IL-1β plasma levels to determine if peripheral inflammation was blocked by MCC950 treatment and investigated its effect on myocardial dysfunction, sublingual microcirculation, 48 h-survival, and neurologic deficits in a rat CA and CPR model. We found that administration of MCC950 following ROSC reduces circulatory levels of IL-1β and cTnI, increases EF and CO, and improves MPI. This secondarily leads to improvement of MAP, ETCO_2_, sublingual microcirculation, and urine output, as well as prolongs survival duration together with better neurologic outcome compared to the control. The favorable effect of MCC950 on neurologic outcome may partly be attributed to the ability of MCC950 to reduce inflammatory responses, which secondarily increases EF and CO. High EF and CO accompanied with high MAP together result in an increase in ETCO_2_ and urine output and the restoration of microcirculatory flow. Improvement of microcirculatory blood flow assists in delivering oxygen and nutrients and the removal of metabolites, oxygen-free radicals, and inflammatory factors. All these profitable factors may ultimately contribute to the improved survival rate and recovery of neurologic function after ROSC. Our current findings indicate that inhibition of NLRP3 with MCC950 may be a new potential therapeutic option to mitigate post-resuscitation myocardial and improve survival and neurologic prognosis.

Coll et al. show that MCC950 prevents NLRP3 inflammasome formation via inhibiting oligomerization of NLRP3-induced apoptosis-associated speck-like protein containing a caspase recruitment domain [14]. In the present study, we also found that MCC950 significantly reduces the expression of ASC, caspase-1 p20, and the production of proinflammatory IL-1β in the myocardium, which are crucial mediators of myocardial injury. MCC950 treated animals have better myocardial function and neurologic outcomes. Thus, the protective effect of MCC950 may partially be attributed to inhibition of NLRP3 inflammasome activation. However, MCC950 had no significant effect on the expression of NLRP3, indicating that MCC950 does not inhibit the priming phase of NLRP3 activation. Coll et al. report that MCC950 directly interacts with the Walker B motif within the NLRP3 NACHT domain, blocking ATP hydrolysis and inhibiting NLRP3 activation and inflammasome formation [32].

There are several limitations of this study. First, healthy animals without underlying disease were included, which is not consistent with clinical conditions. Second, MCC950 was administered by intraperitoneal injection, which would not apply to human victims. Third, histologic analysis to demonstrate attenuation of heart injury was not performed and should be validated in future studies. Fourth, animal size in survival subgroups is insufficient. Further studies with a larger sample size are needed to further confirm the survival results.

## Conclusions

NLRP3 inflammasome activation mediated overproduction of IL-1β is involved in post-resuscitation myocardial dysfunction in a rat model of CA and CPR. Selective inhibition of the NLRP3-inflammasome with MCC950 at ROSC reduces circulatory levels of IL-1β, attenuates post-resuscitation myocardial dysfunction, and improves 48 h-survival rate and neurologic deficit.

## Supplementary Information

Below is the link to the electronic supplementary material.

Supplementary file1 (DOCX 16 KB)

Supplementary file2 (MP4 9468 KB)

Supplementary file3 (MP4 5960 KB)

Supplementary file4 (MP4 4182 KB)

## Data Availability

The data used to support the findings of this study are available from the corresponding authors upon request.
