# Supplementary material for: The Selective NLRP3-inflammasome inhibitor MCC950 Mitigates Post-resuscitation Myocardial Dysfunction and Improves Survival in a Rat Model of Cardiac Arrest and Resuscitation
Source: Cardiovasc Drugs Ther. 2022 Jan 1;37(3):423–33. doi: 10.1007/s10557-021-07282-z (PMC10164003; doi:10.1007/s10557-021-07282-z)
Supplement: Supplementary file 1 — Supplementary file1 (DOCX 16 KB) [file 10557_2021_7282_MOESM1_ESM.docx]

SUPPLEMENTAL MATERIAL

Legends for Video Files:

Supplemental video 1. MCC950 animal survived 48h with good neurologic outcome.

Supplemental video 2. Only control animal that survived 48h with poor neurologic outcome.

Supplemental video 3. Sham animal survived 48h with normal neurologic outcome.

(Videos were captured by the first author during observation of post-resuscitation.
